# Which is the best lipid-modifying strategy in metabolic syndrome and diabetes: fibrates, statins or both?

**DOI:** 10.1186/1475-2840-3-10

**Published:** 2004-12-01

**Authors:** Alexander Tenenbaum, Enrique Z Fisman

**Affiliations:** 1 Cardiac Rehabilitation Institute, the Chaim Sheba Medical Center, 52621 Tel-Hashomer, Ramat-Gan, Israel; 2 Sackler Faculty of Medicine, Tel-Aviv University, 69978 Ramat-Aviv, Tel-Aviv, Israel

**Keywords:** Diabetes mellitus, Dyslipidemia, Fibrates, Metabolic syndrome, Statins

## Abstract

Although less clinical intervention studies have been performed with fibrates than with statins, there are evidences indicating that fibrates may reduce risk of cardiovascular events. The potential clinical benefit of the fenofibrate will be specified by the ongoing Fenofibrate Intervention and Event Lowering in Diabetes (FIELD) study, which rationale, methods and aims have been just published.

Controlled clinical trials show similar or even greater cardiovascular benefits from statins-based therapy in patient subgroups with diabetes compared with overall study populations. Therefore, statins are the drug of first choice for aggressive lipid lowering actions and reducing risk of coronary artery disease in these patients. However, current therapeutic use of statins as monotherapy is still leaving many patients with mixed atherogenic dyslipidemia at high risk for coronary events. A combination statin/fibrate therapy may be often necessary to control all lipid abnormalities in patients with metabolic syndrome and diabetes adequately, since fibrates provide additional important benefits, particularly on triglyceride and HDL-cholesterol levels. Thus, this combined therapy concentrates on all the components of the mixed dyslipidemia that often occurs in persons with diabetes or metabolic syndrome, and may be expected to reduce cardiovascular morbidity and mortality.

Safety concerns about some fibrates such as gemfibrozil may lead to exaggerate precautions regarding fibrate administration and therefore diminish the use of the seagents. However, other fibrates, such as bezafibrate and fenofibrate appear to be safer and better tolerated. We believe that a proper co-administration of statins and fibrates, selected on basis of their safety, could be more effective in achieving a comprehensive lipid control as compared with monotherapy.

## 

Due to their beneficial effects on glucose and lipid metabolism, peroxisome proliferator activated receptors (PPAR's) alpha agonists (fibrates) are good potential candidates for reducing the risk of myocardial infarction (MI) in subjects with metabolic syndrome [1-3]. Although less clinical intervention studies have been performed with fibrates than with statins, there are evidences indicating that fibrates may reduce risk of cardiovascular disease and particularly non-fatal MI [4-10]. Interestingly, reduction of cardiovascular disease with one of the fibric acid derivates – gemfibrozil – was more pronounced in patients displaying baseline characteristics very similar to metabolic syndrome definitions [4,5].

There have been no direct head-to-head comparisons of a statin with a fibrate in any clinical endpoint trial. However, compared with statins, fibrates appear to more selectively target the therapeutic goals in obese individuals with features of insulin resistance and metabolic syndrome (i.e. with near-goal low-density lipoprotein (LDL)-cholesterol and inappropriate high-density lipoprotein (HDL)-cholesterol and triglyceride levels).

The primary-prevention trial Helsinki Heart Study showed that treatment with gemfibrozil led to a significant reduction in major cardiovascular events [4]. Regarding secondary prevention, in the VAHIT study (Veterans Affairs High-density lipoprotein cholesterol Intervention Trial) – which included 30% of diabetic patients – gemfibrozil reduced the occurrence of major cardiovascular events by 22 % [5]. Similarly, reduction of cardiovascular disease with gemfribrozil was more pronounced in patients displaying above three of the features of metabolic syndrome [11,12].

In two previous small studies bezafibrate decreased the rate of progression of coronary atherosclerosis and decreased coronary event rate [6,7]. In another large trial in 1568 men with lower extremity arterial disease with a relatively short follow-up period, bezafibrate reduced the severity of intermittent claudication for up to three years. [8]. Ingeneral, the incidence of coronary heart disease in patients on bezafibrate has tended to be lower , but this tendency did not reach statistical significance. However, bezafibrate had significantly reduced the incidence of non-fatal coronary events, particularly in those aged <65 years at entry, in whom all coronary events may also be reduced [8]. In the Bezafibrate Infarction Prevention (BIP) study an overall trend of a 9.4% reduction of the incidence of primary end point (fatal or non-fatal myocardial infarction or sudden death) was observed. The reduction in the primary end point in 459 patients with high baseline triglycerides (≥200 mg/dL) was significant [9]. These results are consistent with studies in experimental models showing that pre-treatment of rats with the PPAR-alpha agonist clofibrate causes a significant reduction in induced myocardial infarct size of 43% [13].

Recently, reduced incidence of type 2 diabetes in patients with impaired fasting glucose level on bezafibrate has been demonstrated [14]. The potential clinical benefit of the other widespread fibric acid derivative, fenofibrate, on the reduction of cardiovascular disease is still unknown and will be specified by the ongoing Fenofibrate Intervention and Event Lowering in Diabetes (FIELD) study, which rationale, methods and aims have been just published [15]. It will be the largest (approximately 10000 patients) ever conducted fibrate-based controlled clinical trial in diabetic patients. The results are expected for 2005. An added strength of this trial is its ability to examine important clinical outcomes across diverse ethnic and gender subgroups. The results of this study will clarify whether the beneficial lipid-modifying effects of micronised fenofibrate lead to a reduction of cardiovascular morbidity and mortality.

Despite increasing use of statins, a significant number of coronary events still occur and many of such events take place in patients presenting with the metabolic syndrome. Whereas statins remain the drug of choice for patients who need to achieve the LDL-cholesterol goal, fibrate therapy may represent the alternative intervention for subjects with atherogenic dyslipidemia typical for metabolic syndrome and an LDL-cholesterol already close to goal values. In addition, the concomitant use of fibrates seems to be attractive in patients whose LDL-cholesterol is controlled by statin therapy but whose HDL-cholesterol and/or triglyceride levels are still inappropriate [16-19]. This strategy will be tested in the ongoing Action to Control Cardiovascular Risk in Diabetes (ACCORD) trial [20].

The factor that dominates in overweight-related metabolic syndrome is the permanent elevation of plasma free fatty acids (FFA) and the predominant utilization of lipids by the muscle inducing a diminution of glucose uptake and insulin resistance. Currently, an insulin-resistant state – as the key phase of metabolic syndrome – constitutes the major risk factor for development of macrovascular complications [21-23]. On the basis of the current concept of the evolution of adipogenesis via PPAR modulation toward insulin resistance and atherothrombotic macrovascular complications (including MI), the decreasing of plasma FFA and improving of insulin sensitization by PPAR agonists seems to be a logical and valuable goal for therapy.

It is important to note that on a whole-body level, lipid and glucose metabolisms interact intimately. Briefly, PPAR alpha is activated by fibric acids (e.g. bezafibrate) and form heterodimers with the 9-cis retinoic acid receptor (RXR). These heterodimers bind to peroxisome proliferator response elements, which are located in numerous gene promoters and increase the level of the expression of mRNAs encoded by PPAR alpha target genes. Bezafibrate reduces triglyceride plasma levels through increases in the expression of genes involved in fatty acid-beta oxidation and by decrease in apolipoprotein C-III gene expression. Fibric acids increase HDL-cholesterol partly by increasing apolipoprotein A-I and apolipoprotein A-II gene expression. Their triglyceride-lowering and HDL-cholesterol raising effects lead to decreased systemic availability of fatty acid, diminished fatty acid uptake by muscle with improvement of insulin sensitization and reduced plasma glucose level [24-28].

Evidence also suggests that there is a 'fibrate effect' that mediates the reduction in CHD risk beyond the favorable impact of these agents on HDL-cholesterol levels. This last notion is consistent with the pleiotropic effects of fibrates which are known to be related to their mechanisms of action [29]. Being PPAR alpha ligands, fibrates have a significant impact on the synthesis of several apolipoproteins (apo) and enzymes of lipoprotein metabolism as well as on the expression of several genes involved in fibrinolysis and inflammation. Such changes contribute to improve the catabolism of triglyceride-rich lipoproteins, leading to a substantial increase in HDL-cholesterol levels accompanied by a shift in the size and density of LDL particles (from small, dense LDL particles to larger, more buoyant cholesteryl ester-rich LDL).

Controlled clinical trials show similar or even greater cardiovascular benefits from statins-based therapy in patient subgroups with diabetes, impaired fasting glucose, and metabolic syndrome, compared with overall study populations. Therefore, statins are the drug of first choice for aggressive lipid lowering actions and reducing risk of coronary artery disease in these patients. However, current therapeutic use of statins as monotherapy is still leaving many patients with mixed atherogenic dyslipidemia at high risk for coronary events.

A combination statin/fibrate therapy may be often necessary to control all lipid abnormalities in patients with metabolic syndrome and diabetes adequately, since fibrates provide additional important benefits, particularly on triglyceride and HDL-C levels. Thus, this combined therapy concentrates on all the components of the mixed dyslipidemia that often occurs in persons with diabetes or metabolic syndrome, and may be expected to reduce cardiovascular morbidity and mortality.

Safety concerns about some fibrates such as gemfibrozil may lead to exaggerate precautions regarding fibrate administration and therefore diminish the use of the seagents. However, other fibrates such as bezafibrate and fenofibrate appear to be safer and better tolerated [30-36]. We believe that a proper co-administration of statins and fibrates, selected on basis of their safety, could be more effective in achieving a comprehensive lipid control as compared with monotherapy.

## Competing interests

The author(s) declare that they have no competing interests.
